# Toward the Relevance of Platelet Subpopulations for Transfusion Medicine

**DOI:** 10.3389/fmed.2018.00017

**Published:** 2018-02-05

**Authors:** Stefan Handtke, Leif Steil, Andreas Greinacher, Thomas Thiele

**Affiliations:** ^1^Institut für Immunologie und Transfusionsmedizin, Greifswald, Germany; ^2^Interfakultäres Institut für Funktionelle Genomforschung, Greifswald, Germany

**Keywords:** platelet subpopulation, platelet size, platelet turnover, platelet clearance, platelet maturation

## Abstract

Circulating platelets consist of subpopulations with different age, maturation state and size. In this review, we address the association between platelet size and platelet function and summarize the current knowledge on platelet subpopulations including reticulated platelets, procoagulant platelets and platelets exposing signals to mediate their clearance. Thereby, we emphasize the impact of platelet turnover as an important condition for platelet production *in vivo*. Understanding of the features that characterize platelet subpopulations is very relevant for the methods of platelet concentrate production, which may enrich or deplete particular platelet subpopulations. Moreover, the concept of platelet size being associated with platelet function may be attractive for transfusion medicine as it holds the perspective to separate platelet subpopulations with specific functional capabilities.

## Introduction

Platelets recognize vessel damage, trigger coagulation and enhance clot formation at the site of injury (1). Beyond hemostasis, platelets also act as mediators in immunity and inflammation (2–5).

Circulating platelets differ in age, maturation state, or density. An obvious physical feature of platelets is their size, which can vary substantially among platelets of one individual. It was an early concept, that large platelets represent a rather young and reactive platelet subpopulation (6). Later, this concept was abandoned when consecutive experiments demonstrated no clear correlation between platelet size and age (7, 8).

The observation that some platelets have particular procoagulant capabilities led to the concept of platelet subpopulations with different biological functions (9). Other examples for platelet subpopulations are reticulated (rather young) platelets and platelets exposing signals mediating their clearance from the circulation (rather old platelets). It is conceivable albeit unclear, whether other platelet subpopulations exist which play a more pronounced role in immunological or inflammatory processes, e.g. by expression of CD40 or release of CD40L (10, 11).

Epidemiological studies found an association between an increased platelet size and thrombotic outcomes in patients with cardiovascular disease (12) resulting in a revival of the “old” hypothesis of an association between a larger platelet size and enhanced platelet function in hemostasis.

Clarifying the hypothesis of different biological features of platelet subpopulations is potentially relevant for transfusion medicine. Enrichment of distinct platelet subpopulations in platelet concentrates (PCs) during production may modulate the biological effects of PCs.

In this review, we summarize the current knowledge on platelet subpopulations with a special emphasis on platelet size, its association with platelet function and the impact of platelet turnover on platelet production.

## Size as a Platelet Characteristic

### Platelet Formation, Turnover, and their Role for Platelet Size

Platelet size is genetically determined and relatively stable over the lifetime in healthy individuals. Genome wide association studies in healthy subjects identified several genes associated with platelet size (13–18).

Under steady-state conditions, platelets are generated from megakaryocytes in the bone marrow after stimulation with thrombopoietin. The amount of circulating thrombopoietin is regulated by the mass of circulating platelets. They bind thrombopoietin, providing a negative feedback mechanism to control thrombopoiesis (19). In mice, thrombopoietin administration increases platelet size (20) whereas in humans the opposite seems to be the case (21).

In the bone marrow, preplatelet intermediates are formed as extensions of elongated megakaryocyte-pseudopodia and released into the sinusoidal blood vessels (22, 23). Glycoprotein Ib mediates transmigration of megakaryocytes into the sinusoids *via* the small GTPases Cdc42 and RhoA (24). Preplatelets convert into barbell-shaped proplatelets that form platelets (23, 25) mediated by integrin αIIbβIII signaling (26). Platelet size is established during the formation of barbell proplatelets from circular preplatelets and limited by microtubule bundling, elastic bending, and actin-myosin-spectrin cortex forces (27).

Thrombopoiesis in the bone marrow is spatially regulated (28) but platelet maturation does not end in the bone marrow. Preplatelets are also formed from proplatelets in the circulation (29) and can maturate in the lungs (30).

*In vivo*, the mechanisms of proplatelet formation are very dynamic and influenced by platelet turnover (31). In case of inflammation an alternative pathway of platelet production can occur. Nishimura et al. found that increased serum levels of the inflammatory cytokine IL-1α induce platelet release by the rupture of megakaryocytes as a distinct mechanism in the absence of elevated thrombopoietin (32). *Via* this mechanism, larger platelets are produced than in thrombopoietin-stimulated megakaryocytes in mice.

We have established a model of enhanced platelet production in healthy volunteers using platelet apheresis showing that platelet apheresis stimulated platelet production leads to reversible changes in the platelet proteome (33). This further indicates an impact of platelet turnover on the phenotype of circulating human platelets.

### Platelet Size and Function during Steady-State Platelet Production

Most studies identified large platelets as a subpopulation with a higher prohemostatic capacity, if generated under steady state. However, it is still debated whether a larger size alone contributes to this higher capacity (34), or if there are specific features in large platelets which over-proportionally increase their prohemostatic potential. Table 1 provides an overview of functional comparisons between large and small human platelets. The majority of experiments included adjustments for cell size, suggesting a hyperproportional prohemostatic capacity of large platelets.

**Table 1:** Functional characterization of human large and small platelets.

Reference	Results	Size adjustment	Evidence for a hyperproportional difference between large and small platelets
**Steady-state platelet production**			
Booyse et al. (35)	Only large platelets contain ribosomes	Not performed	Yes

Karpatkin (36)	Large platelets: higher glycogen, higher orthophosphate, higher total adenine nucleotide, higher glucogenolysis capacity, higher glycolysis activity, higher protein synthesis, higher glycogen synthesis, higher resistance to osmotic shock	Ratios of analytes compared to ratios of platelet volumes	Yes

Karpatkin (37)	Large platelets: lag time to aggregation shorter; higher ATP release; following aggregation higher ADP release; higher release of platelet factor 4	Not performed	Not applicable

Karpatkin and Strick (38)	Large platelets: higher activity of glycolysis enzymes, less lipid peroxidation product, more resistant to lipid peroxidation	Equal amount of protein extract taken from large and small platelets	Yes

Thompson et al. (34)	Large platelets: maximal aggregation after activation by collagen or thrombin increased; contain larger amounts of ATP and beta-thromboglobulin	Relative change within each size fraction (aggregometry); relative comparison of ATP and beta-thromboglobulin before and after stimulation	Yes

Jakubowski et al. (39)	Large platelets: release more thromboxane after collagen or thrombin stimulation	Correlation to MPV	No
	Platelet size correlates with the amount of metabolized arachidonic acid		

Mezzano et al. (40)	Large platelets: more fibrinogen, more serotonin and more absolute protein	Not performed	Not applicable

Pereira et al. (41)	Large platelets: more P1^a1^ molecules; small platelets: more HLA-A2 molecules, more total HLA class I-molecules	Not performed	Not applicable

Frojmovic et al. (42)	Large platelets: more fibrinogen receptor expressed on membrane when activated; faster aggregation rate	Correlation of ratios large/small with size ratio large/small	No

Polanowska-Grabowska et al. (43)	Large platelets: faster adhesion to collagen, less sensitive to inhibition by prostacyclin, increased content of glycoprotein Ia/iia complex	Not performed	Not applicable

Opper et al. (44)	Large platelets: higher basal level of cgmp, higher cgmp synthesis rate after stimulation with sodium nitroprusside, lower activity of camp-dependent phosphodiesterases	Adjustment of protein content and platelet size	Yes

Li et al. (45)	Large platelets: higher maximal aggregation after stimulation with thrombin, increased ATP secretion, higher degree of calcium mobilization	Relative change within each size fraction (aggregometry)	Yes

Opper et al. (46)	Large platelets: higher degree of protein phosphorylation after thrombin stimulation, higher rate of ADP-ribosylation by cholera toxin; small platelets: higher basal phosphorylation levels of several proteins, higher ADP-Ribosylation by pertussis toxin and C3 exoenzyme, higher basal Ca^2+^-level	Equal amount of protein extract taken from large and small platelets	Yes

Mangalpally et al. (47)	Large platelets: express more surface-bound fibrinogen, bind more von Willebrand factor after arachidonic acid- or ADP-stimulation, express more P-selectin, more activated glycoprotein iib/iiia after ADP stimulation; higher proportion of reticulated platelets	Adjustment to the platelet surface area	Yes

Brambilla et al. (48)	Large platelets: contain higher amounts of tissue factor and tissue factor mrna; mainly large platelets expose functionally active tissue factor	Not performed	Not applicable

**Increased platelet turnover**			
Balduini et al. (49)	Old platelets: MPV and P-LCR reduced; young platelets: MPV and P-LCR higher compared to old and to control; aggregation response faster in young platelets	Relative change within each size fraction (aggregometry)	Yes

*MPV, mean platelet volume; P-LCR, platelet large cell ratio; HLA, human leukocyte antigen*.

Steady-state large platelets have a higher capacity for glucose metabolism, resistance to osmotic shock (36), and lipid peroxidation (38). They aggregate faster and release more ATP and alpha granule proteins (34, 37), contain more fibrinogen, and serotonin (40), and express more human leukocyte antigen-I molecules (41) and membrane glycoproteins (43).

Platelets synthesize proteins (50) and large platelets have more ribosomes and incorporate more amino acids (35). Probably, large platelets have a higher capacity to translate mRNA. This needs to be demonstrated by future studies, which adequately control for residual leukocytes in the large platelet fraction.

Opper et al. found different patterns of cGMP synthesis and protein phosphorylation patterns after stimulating platelets of different size (44, 46), suggesting differences in signal transduction between large and small platelets.

The ability to mobilize Ca^2+^ in the cytosol is pivotal for platelet activation. Li et al. showed that the cytosolic Ca^2+^-concentration is similar in resting large and small platelets, whereas higher amounts of Ca^2+^ are mobilized by large platelets (45).

Large platelets express more surface-bound fibrinogen, bind more von Willebrand factor, and metabolize more arachidonic acid (39), express more P-selectin, activate more integrin αIIbβ3 after ADP-stimulation (42, 47, 51), and release more thromboxane after collagen- and thrombin-induced aggregation in proportion to platelet size (39).

A recent study indicates that large-size platelets are functionally different compared to small platelets. Brambilla et al. found that large platelets express not only significantly higher amounts of tissue factor and tissue factor mRNA compared to small platelets. Large platelets also expose functionally active tissue factor on their cell membranes whereas the activity of tissue factor in small platelets is almost completely quenched by tissue factor pathway inhibitor (48). These results extent previous findings showing that platelets translate tissue factor (52) and point toward specific roles of large and small platelets in hemostasis.

### Platelet Size during Increased Platelet Turnover

If platelet production is enhanced in healthy humans by application of thrombopoietin, the peripheral platelet concentration increases whereas platelet size measured by the mean platelet volume (MPV) slightly decreases without changes in platelet viability, platelet responsiveness to physiologic agonists, or expression of platelet activation markers (21).

In contrast, disease-related increased platelet turnover is often associated with an increase in platelet size (6, 53), e.g., in case of enhanced destruction of platelets by autoantibodies (54–56), during recovery after bone marrow suppression by chemotherapy (49), or in situations with increased consumption in patients with severe arterial disease (57, 58). These studies suggest that platelet size in disease is regulated by other mechanisms than the ones regulating platelet size during thrombopoietin-mediated megakaryocytopoiesis in healthy volunteers. Severe thrombocytopenia induced by disseminated intravascular coagulation in children is also associated with an increase in platelet size (59). However, in view of the findings of Nishimura et al. (32), this likely results from platelet production by the alternative pathway involving IL-1α induced fragmentation of megakaryocytes.

### Platelet Size and Platelet Age

The first attempts to characterize young platelets were driven by the hypothesis, that large platelets are considerably younger than small platelets because they are more functionally active (37, 38). However, later studies did not reveal a direct relationship between platelet size and age. This was convincingly underscored by an experiment in baboons under conditions of steady-state platelet production. The animals received radioactively labeled methionine being incorporated by megakaryocytes (8). Radioactively labeled platelets were afterwards present in each assessed size fraction of platelets indicating that size and age of platelets do not correlate under steady-state conditions. Also in humans platelet size is likely not strongly associated with platelet age (7). After transfusion of radioactively labeled autologous platelets, the mean survival of a high-density platelet population was shorter than that of platelets with low density. The mean volumes of high- and low-density platelets were not different suggesting that platelet size is unrelated to platelet age under normal conditions, but implicating a role of platelet density for the age of circulating platelets.

### Platelet Size as Risk Factor for Adverse Clinical Outcomes

Epidemiological studies in patients with cardiovascular disease found an association between an increased MPV and a higher prevalence of thromboembolic complications (12, 60–62). An increased platelet size due to increased platelet turnover also correlates with refractoriness to antiplatelet therapy (58) and predicts a higher incidence of adverse outcomes after coronary intervention (63).

It is unclear, whether the increased MPV is the cause or the consequence of an increased risk for thromboembolic outcomes (60, 64). An alternative explanation is that individuals with large platelets have *per se* an increased risk for thrombotic complications because genetic traits have been identified, which are at the same time associated with an increased MPV and an increased risk for cardiovascular disease (65).

An increased MPV also characterizes inherited bleeding disorders with dysfunctional large platelets (66).

## Platelet Subpopulations

### Reticulated Platelets

Ingram et al. first observed a unique population of newly formed platelets soon after the induction of acute blood loss in beagle dogs. They stained platelets with methylene blue and noticed coarse and punctate condensations in platelets similar to those seen in reticulocytes of red cells. Therefore, this platelet fraction was named “reticulated platelets” (67). Later, reticulated platelets were shown to contain more RNA, staining with nucleic acid-specific fluorescent dyes, such as thiazole orange (68).

Reticulated platelets likely represent the youngest platelet fraction. After *in vivo* biotinylation, freshly formed platelets carrying reduced levels of biotin were shown to be reticulated (69). These platelets are younger than 24 h (70) and decay their RNA during aging (71).

In healthy humans with steady-state platelet production around 8% of circulating platelets are reticulated (72). Furthermore, the proportion of reticulated platelets is enriched in the fraction of large platelets compared to the fraction of small platelets (47), suggesting a relationship between platelet size and age.

A limitation of studies applying thiazole orange to stain reticulated platelets is, however, the tendency of this dye to bind unspecifically to alpha-granule contents (73). Therefore, a higher proportion of thiazole orange positive platelets observed in larger platelets could in part result from unspecific binding and may not represent young platelets (74). This limitation may be overcome by more RNA-specific dyes (75), which may finally elucidate the relationship between the size and age of platelets under steady-state platelet production.

In patients with high platelet turnover, the MPV is increased and likewise the proportion of reticulated platelets (72, 76, 77). One example that these changes may have biological relevance is their response to antiplatelet therapy. Despite dual antiplatelet therapy, large platelets with a higher proportion of reticulated platelets show increased *in vitro* reactivity compared to small platelets (78). Moreover, newly formed reticulated platelets show increased thrombogenicity after stopping prasugrel (75). Both observations suggest consequences for individualized antiplatelet therapy.

### Procoagulant Platelets

About 30% of circulating platelets (range of 15–55%) can exhibit a procoagulant phenotype after stimulation with the agonists *co*llagen *a*nd *t*hrombin (79, 80). They were named COAT-platelets, which was later changed to coated platelets. Coated platelets express high levels of functional α-granule derived Factor V (FV) (79) and other α-granule proteins on their surface, including fibrinogen, von Willebrand factor, thrombospondin, fibronectin, and α_2_-antiplasmin (81). Furthermore, coated platelets expose procoagulant phosphatidylserine (PS) on their surfaces (79, 82). PS exposure on the outer platelet membrane is closely related to disruption of inner mitochondrial membranes in the cells (83). In platelets, this process is controlled by calpain and not by caspases as in other cells (84). Therefore, PS exposure on procoagulant platelets is not necessarily related to apoptosis (85, 86). As not all PS-exposing platelets show the typical features of coated platelets, coated platelets seem to represent a procoagulant subgroup of PS-exposing platelets (82, 87).

Activation of the protease activated receptor 1 with thrombin, SFLLRN, and AYPGKF had strong additional effect (80) on the collagen-induced calcium peak and induced a sustained cytoplasmatic elevation of Ca^2+^ which is crucial for the formation of procoagulant platelets (88). Differential phosphorylation of PKCalpha and p38MAPK may drive the different calcium fluxes in coated compared to non-coated platelets (89). Increased cytosolic Ca^2+^ levels result in the inactivation of adenylatecyclase and activation of phosphatidylinositol 3-kinase and Src tyrosine kinase which further promotes procoagulant platelet segregation (90). On the other hand, elevated cytosolic Ca^2+^ levels can reverse integrin αIIbβ3 activation by stimulating intracellular cleavage of the β3-chain *via* calpain (91). PAC-1 binding is reduced in coated platelets although surface expression of αIIbβ3 is not diminished (89). The underlying mechanism is displacement of PAC-1 a stronger bond rather than inactivation of αIIbβ3 (92). This may explain why coated platelets do not take part in the formation of aggregates mediated by αIIbβ3.

Thus, platelet subpopulations arrange differently in a thrombus (93). Within a thrombus platelets with activated αIIbβ3 integrins assemble to aggregates. Those with inactive αIIbβ3 integrins remain solitary and form blebs and shed microparticles (93–95), the typical features of coated platelets. Independently of αIIbβ3, coated platelets attach to aggregates by forming caps of colocalized fibrinogen and thrombospondin on the PS-positive platelet surface (96). This allows coated platelets to become incorporated into thrombi independently of activated αIIbβ3 integrins.

Interestingly, platelet size has not yet been directly investigated as a feature of procoagulant human platelets. In rabbits, young platelets showed a similar size and the same ability to form procoagulant platelets under steady state compared to older platelets (74). If size is associated with the procoagulant capability of human platelets, it could be applied to enrich or deplete procoagulant platelets in PCs.

### Platelets Exposing Signals for Clearance

Platelets survive for up to 10 days under normal conditions (97, 98). Platelets exposing signals to induce their clearance may be seen as another subpopulation with a limited life span. It would be desirable to reduce the amount of these platelets in PCs to prolong survival of transfused platelets.

Three main mechanisms have been identified by which platelets mediate their clearance (99). First, degraded glycans appear as a signal on platelet membranes which are recognized by the hepatic Ashwell Morrel Receptor (100). This has been demonstrated for cold stored platelets (101) and for platelets in sepsis (102, 103). Concomitantly, the removal of glycan deprived platelets *via* the Ashwell Morell Receptor in the liver induces hepatic thrombopoietin-mRNA expression and leads to increased megakaryocyte numbers and *de novo* platelet production (100).

The second mechanism is platelet apoptosis. Platelet survival is extended if the proapoptotic proteins Bak and Bax are lacking and reduced if the prosurvival proteins Bcl-2, Bcl-xL, and Mcl-1 are absent (104). Recently, protein kinase A was identified as a mediator of platelet life span by regulating apoptosis (105). However, the exact signals on the platelet surface and the corresponding receptor recognizing apoptotic platelets for platelet clearance are not yet identified. It is also unknown whether apoptotic signals appear differently in platelets of different size.

Finally, platelets are cleared after being opsonized with antibodies, which can be autoantibodies in diseases such as autoimmune thrombocytopenia, or alloantibodies in case of feto-maternal incompatibility, or after platelet transfusion (106). This mechanism is likely independent of platelet size.

Of note, P-selectin is an adhesion receptor for leukocytes expressed by activated platelets and was suggested to mediate platelet clearance. Berger et al. demonstrated that P-selectin does not mediate platelet clearance but may modulate leukocyte recruitment or thrombus growth (107).

## Conclusion and Perspectives

Understanding features differentiating platelet subpopulations has greatly improved. For example, platelet size correlates with platelet reactivity and mRNA content, which may classify large platelets as a prohemostatic subpopulation. These large platelets could be enriched in blood centers by differential or density gradient centrifugation, or special apheresis techniques in order to produce more potent PCs, e.g., for trauma patients.

It remains unclear, if large platelet fractions also include more procoagulant platelets. To gain further insight, PS-exposure, Ca^2+^-mobilization and the ability to form coated platelets should be assessed in large and small platelets. Additionally, no data exist whether immunological functions of platelets correlate with platelet size.

Highly relevant for the interpretation of any study on the association of platelet size and platelet function is the fact that platelet turnover is important for platelet formation. Large platelets under steady state are likely different from large platelets generated under conditions of increased platelet turnover. This difference may explain some of the conflicting results on large and small platelets reported in the literature. It will be mandatory for future studies to exactly define the conditions of platelet turnover under which the investigated platelet population is generated as well as the agonists mediating thrombopoiesis in health and disease (108).

Platelet turnover may also be relevant for the production of PCs. Platelets derived from whole blood donation are collected under steady-state conditions because the donation procedure routinely lasts ~5–15 min and will unlikely result in changes in the collected platelets. In contrast, repeated platelet apheresis procedures may stimulate platelet generation because it lowers the platelet content more rapidly over a period of 60–90 min (109) and can be performed up to 3 times a week. This may have an effect on the collected platelet population, as shown for the platelet proteome after repeated apheresis (33). Moreover, platelets collected from hypertensive donors may differ in phenotype and functionality compared to those from normotensive donors (110).

Finally, PCs are produced by differential centrifugation leading to a loss of very large and very small platelets. Recently it was shown that the preparation procedure of red cell concentrates is associated with mortality (111). Enrichment of a specific platelet subpopulation in PCs by different preparation methods might also be relevant for the outcomes of transfused patients.

In summary, there is increasing evidence on platelet subpopulations with different biological functions, which are particularly interesting for transfusion medicine. Better understanding of the characteristics and functions of platelet subpopulations may be applied to develop new or improved platelet products.

## Author Contributions

All authors listed have made a substantial, direct, and intellectual contribution to the work and approved it for publication.

## Conflict of Interest Statement

The authors declare that the research was conducted in the absence of any commercial or financial relationships that could be construed as a potential conflict of interest.
